# Measurement equivalence of the English, Chinese and Malay versions of the World Health Organization quality of life (WHOQOL-BREF) questionnaires

**DOI:** 10.1186/s12955-019-1130-0

**Published:** 2019-04-17

**Authors:** Yin Bun Cheung, Khung Keong Yeo, Kok Joon Chong, Eric Yin Hao Khoo, Hwee Lin Wee

**Affiliations:** 1 Centre for Quantitative Medicine, Duke-NUS Medical School, Singapore, Singapore; 2 Tampere Center for Child Health Research, University of Tampere and Tampere University Hospital, Tampere, Finland; 3 National Heart Centre Singapore, Singapore, Singapore; 4 Duke-NUS Medical School, Singapore, Singapore; 5 Department of Medicine, Yong Loo Lin School of Medicine, National University of Singapore, Singapore, Singapore; 6 Division of Endocrinology, University Medicine Cluster, National University Hospital, Singapore, Singapore; 7 Department of Pharmacy, Faculty of Science, National University of Singapore, Singapore, Singapore; 8 Saw Swee Hock School of Public Health, National University of Singapore, Singapore, Singapore

**Keywords:** Quality of life, Equivalence, Patient-reported outcomes, Preference-based measures

## Abstract

**Background:**

The WHOQOL-BREF is a widely used questionnaire for measuring quality of life. It is important to establish the measurement equivalence of various language versions of WHOQOL-BREF so that scores from different language versions may be pooled together. The primary aim of this article was to evaluate the measurement equivalence of the English, Chinese and Malay versions of the WHOQOL-BREF.

**Methods:**

We analysed data from the previously published, cross-sectional, WONDERS study and used linear regression models to adjust for potential confounding variables. Based on equivalence clinical trial methods, measurement equivalence was assessed by comparing 90% confidence interval (CI) of differences in scores across language versions with a predefined equivalence margin of 0.3 SD. Equivalence was achieved if the 90% CI fell within 0.3 SD. Data from 1203 participants, aged above 21 years, were analysed.

**Results:**

Participants who completed the different language versions of WHOQOL-BREF expectedly differed in age, ethnicity, highest education level, marital status, smoking status and Body Mass Index (BMI). The English and Malay language versions were definitely equivalent for all domains. The English and Chinese language versions were definitely equivalent for physical and environmental domains but inconclusive for psychological and social domains. Likewise, for Chinese and Malay versions.

**Conclusion:**

The English, Chinese and Malay language versions of the WHOQOL-BREF questionnaire may be considered equivalent, with evidence being more robust for some domains than the others. Given the large number of people who speak/ read Chinese and Malay, this study has widespread relevance.

## Background

Patient reported outcomes (PRO) are reports obtained directly from patients about how they function or feel in relation to a health condition and its therapy, without interpretation of the patient’s response by a clinician or anyone else [1]. PRO are used in clinical trials to evaluate the effectiveness of medical interventions from the patients’ perspective [2], as a surrogate measure of the direct benefit to the patient’s well-being [3]. The results of these studies inform healthcare decisions made by patients and their clinicians, influence the development of health policy and support licensing claims for medication [2].

World Health Organization Quality-of-Life Questionnaire (WHOQOL-BREF) is a health-related quality of life (HRQoL) assessment used as a PRO. The WHOQOL-BREF, an abbreviated version of WHOQOL-100, was developed as a cross-cultural questionnaire and is available in more than 20 languages [4]. It has since been used in many countries including United States of America [5, 6], Thailand [7, 8], India [9, 10], China [11, 12], Ghana [13, 14] and New Zealand [15, 16]. In multinational clinical trials and trials within multi-ethnic societies, it is common to use multiple language versions of PRO questionnaires to cater to the different language needs of the study participants. Various language versions of the questionnaires are combined in order to increase the power and representativeness of the study. To do so, a pre-requisite is that the language versions are considered equivalent in terms of their measurement properties. [1] This is important as culture and language could affect the respondents’ interpretation and responses to the HRQoL instruments. For example, in a study among students from Flanders, Belgium and Iran, [17] the authors reported that participants seem to respond differently to several items in the WHOQOL-BREF, especially those items in the physical and psychological domains. In another example, it was reported that the scores of one-third of the facets and domains in the WHOQOL-BREF were significantly different between the English and Hindi versions even though the questionnaires were completed by participants who are bilingual in English and Hindi. [18] Hence, the aim of this study is to evaluate the equivalence of the English, Chinese and Malay versions of the WHOQOL-BREF in Singapore.

Singapore is a multi-ethnic country, with Chinese, Malays and Indians making up the majority of the population. Although English is the main language of commerce and education, there is a sizeable proportion of the population who are more comfortable with their mother tongue languages [19, 20]. The psychometric properties of the Tamil version of the WHOQOL-BREF have not been evaluated in Singapore. Hence, we did not use the Tamil version in our study. In addition, English, Chinese and Malay are also commonly spoken languages in other South East Asian countries such as Malaysia, the Philippines and Indonesia. [21] Furthermore, Standard Mandarin is spoken by a large number of people in China, Chinese Taipei, Malaysia and Indonesia. [22, 23]

## Methods

### Participants and study design

This study comprises of participants from the general population as well as two clinic samples. Ethics approval for this study was obtained from the National Healthcare Group Domain Specific Review Board (Ref. 2013/00747) as well as the SingHealth Centralised Institutional Review Board (Ref. 2015/2041). The clinic samples were chosen to enrich the dataset such that a wider spread of health status was represented, and the generalizability of the findings will be enhanced.

The general population participants were recruited by using a multi-stage cluster sampling using postcodes as the primary sampling unit (PSU) followed by the selection of household then the selection of respondents. Three call attempts (1st attempt and 2 call backs) were made at different days and different times of the week. Only one participant per household was selected. Participants in each contacted household were selected based on a pre-specified quota for language of interviews within each ethnicity interlocking with age and gender. The face-to-face interviews were conducted in the participants’ home between October 2014 to January 2015.

The clinic participants were drawn from two separate studies in outpatient clinics in the National Heart Center Singapore (NHCS) and the Division of Endocrinology in the National University Hospital (NUH). Recruitment was conducted by research assistants via convenience sampling in the clinics while patients were waiting to see the doctor. In both clinical samples, patients must respond positively to either of these questions “Have you ever been told by a doctor previously that you have at least one of the following: blockage of the arteries to your heart, stroke, heart attack, peripheral arterial disease, or transient ischaemic attack, or kidney disease.” and “Have you undergone at least one of the followings: heart bypass operation, stent insertion or brain surgery for stroke”. Patients with recent acute myocardial infarction (STEMI), hemodynamic instability or gestational diabetes were excluded. Interviews were carried out between March 2015 and February 2016.

In both general population and clinic samples, to be eligible, the participant must be a Singapore Resident (including Singapore Citizens and Singapore Permanent Residents) aged 21 years and above who speaks English, Chinese (Mandarin) or Malay. Participants who speak only Tamil were excluded as the psychometric properties of the Tamil version of the WHOQOL-BREF have not been evaluated in Singapore. All participants read and signed the written informed consent form prior to commencement of the interviews. In Singapore, among the resident population aged 15 and over who are literate (95.9%), 78.6% speaks either English only (11.1%) or English and at least one other language (67.5%). English is the first language in school for all Singaporeans since 1959 [24].

### WHOQOL-BREF

The WHOQOL-BREF was administered together with a socioeconomic and clinical questionnaire where information such as age, gender, ethnicity and self-reported medical conditions were captured. The WHOQOL-BREF is a 26-item questionnaire which includes one item from each of the 24 facets contained in the WHOQOL-100 and two additional items on overall quality of life and general health. The 24 items are organized into 4 domains, namely Physical Health, Psychological, Social Relationships and Environment. Three negatively phrased items were reversed scored. According to the user manual, domain scores were computed by taking the mean of the scores of the items that constitute the domain and multiplied by 4 so that the scores are directly comparable with those derived from WHOQOL-100. Mean substitution was performed for missing data by replacing the missing items with the mean of the non-missing item scores in the same domain if there was no more than one missing value per domain. The three language versions are official versions developed by the WHOQOL group.

### Statistical analyses

Participants with more than 20% missing data were excluded from the analysis. Participants with missing data on participant characteristics were also excluded. Participants who completed the different language versions are expected to differ in demographic and socioeconomic characteristics, in particular, educational levels. Hence, we compared the participant characteristics using analysis of variance (ANOVA) for continuous variables such as age and chi-square test for categorical variables such as educational levels. We performed multiple linear regression analyses to compare the WHOQOL-BREF domain and total scores across the three language versions with the English version as the reference with adjustment for age, sex, education, marital status, smoking status, chronic disease and BMI. We entered the variables with a priori specification rather than stepwise regression. This strategy is more appropriate than other variable selection strategies when there is prior knowledge [25]. We did not adjust for ethnicity due to collinearity with language version. In addition, we did not adjust for multiple comparisons as Rothman has previously pointed out that doing so would reduce the type I error for null associations at the expense of increasing the type II error for those associations that are not null. [26, 27]

In the multiple linear regression analyses, we compared the 90% confidence interval (CI) of the betas for the language variable and declared either the Chinese or the Malay version to be equivalent to English version if the 90% CI falls completely within a pre-defined equivalence margin, to be non-equivalent if the 90% CI falls completely outside the pre-defined equivalence margin or inconclusive if the 90% CI overlaps with the equivalence margin [28, 29]. This approach is similar to that of clinical trials that seek to demonstrate the equivalence of two treatments. The use of *p*-values to test for difference suffers the limitation of “no evidence of difference” does not provide “evidence of no difference”. In contrast, the approach of using CI does provide evidence of no difference. Hence, the CI approach is preferred to the p-value approach. There are at least two schools of thoughts with regards to what magnitude of change score in HRQoL is considered a minimally important difference. Norman and colleague suggested that the minimally important difference (MID) is half a standard deviation (SD) [30] while Farivar et al. suggested that the MID is probably 0.3 SD [31]. In our paper, we took the more conservative approach and defined the equivalence margin of difference in scores due to language version to be ±0.3 of a SD. In order to rule out unobserved cultural differences between ethnic groups, we did another set of multiple linear regression analyses, including only ethnic Chinese and ethnic Malay in the English vs Chinese and English vs Malay language version comparisons, respectively.

Based on a mean WHOQOL total score of approximately 75 and SD of 20 points observed in our dataset across the three language versions, we estimated that 175 participants are required in each group to detect a 6-point difference (i.e. 0.3 SD). This manuscript is a secondary analysis of the WONDERS study [32]. The main objective of the WONDERS study was to develop a mapping algorithm between WHOQOL-BREF and EQ-5D-5 L. [33] The WONDERS study recruited 428, 427 and 348 participants who completed the English, Chinese and Malay versions, respectively. Hence, we are adequately powered to perform the proposed analyses.

## Results

### Participant characteristics

There were 428, 427 and 348 participants who completed the English, Chinese and Malay versions of the study questionnaires, respectively. Table 1 shows the participant characteristics by the language version of the WHOQOL-BREF questionnaire. Participants who completed the English version have the lowest mean (SD) age compared to those who completed the Chinese (52.8 (15.6) years old) or Malay (50.7 (14.7) years old) versions. Those who completed the Chinese version were more likely to be older, married, non-smoker and had a lower average BMI. In addition, those who completed the Malay version tended to have lower education and have a higher average BMI. There was no significant difference in sex and presence of chronic disease across the language versions.

**Table 1:** Participant characteristics by language version of WHOQOL-BREF questionnaire

Characteristics	Category	Language			*P*-value^a^
		English (*n* = 428)	Chinese (*n* = 427)	Malay (*n* = 348)	
Age, mean (SD)	–	48.0 (16.5)	52.8 (15.6)	50.7 (14.7)	< 0.001
Sex, *n* (%)	Female	165 (38.6)	184 (43.1)	151 (43.4)	0.288
Ethnicity, *n* (%)	Chinese	172 (40.2)	427 (100.0)	0 (0.0)	< 0.001
	Malay	114 (26.6)	0 (0.0)	345 (99.1)	
	Indian	142 (33.2)	0 (0.0)	3 (0.9)	
Education, *n* (%)	Primary or below	39 (9.1)	127 (29.7)	90 (25.9)	N/A
	Secondary	219 (51.2)	178 (41.7)	222 (63.8)	
	Post-secondary	170 (39.7)	122 (28.6)	36 (10.3)	
Marital status, *n* (%)	Never married	96 (22.4)	63 (14.8)	42 (12.1)	0.002
	Married	302 (70.6)	327 (76.6)	265 (76.2)	
	Divorced/separated	14 (3.3)	19 (4.5)	18 (5.2)	
	Widowed	16 (3.7)	18 (4.2)	23 (6.6)	
Smoking, *n* (%)	Non-smoker	293 (68.5)	316 (74.0)	214 (61.5)	0.007
	Ex-smoker	58 (13.6)	49 (11.5)	61 (17.5)	
	Current smoker	77 (18.0)	62 (14.5)	73 (21.0)	
Chronic disease, *n* (%)	Yes	211 (49.3)	236 (55.3)	189 (54.3)	0.177
Body Mass Index (kg/m^2^), mean (SD)	–	25.4 (4.9)	24.3 (4.1)	27.1 (5.9)	< 0.001

^a^Difference in continuous variables tested by ANOVA; differences in categorical variables tested by Chi-square

### Influence of language version on WHOQOL-BREF domain scores

After adjusting for known determinants of the WHOQOL-BREF, confirmed equivalence of the English and Chinese WHOQOL-BREF language versions was demonstrated for two of the domains, namely physical and environmental while inconclusive equivalence was observed for two other domains (Table 2). The same observations were made when comparing the Chinese and Malay language versions. Confirmed equivalence between the English and Malay language versions was demonstrated for all four WHOQOL-BREF domains.

**Table 2:** WHOQOL-BREF scales: mean (SD) by language version, equivalence margin and adjusted difference between the two language versions

Scale	English (*n* = 428)	Chinese (*n* = 427)	Malay (*n* = 348)	Equivalence Margin^a^	English vs Chinese Difference^b^ (90% CI)	English vs Malay Difference^b^ (90% CI)	Chinese vs Malay Difference^b^ (90% CI)
	Mean (SD)	Mean (SD)	Mean (SD)				
Physical	74.6 (15.8)	71.7 (15.7)	72.3 (18.9)	±5	−1.4 (−3.1 to 0.3)^†^	−0.4 (− 2.2 to 1.3)^†^	− 1.0 (− 2.7 to 0.8)^†^
Psychological	72.3 (14.3)	67.2 (16.0)	70.6 (14.0)	±5	−4.1 (−5.7 to − 2.5)^‡^	− 0.5 (− 2.2 to 1.3)^†^	−3.6 (− 5.4 to − 1.9)^‡^
Social	73.5 (16.2)	68.9 (13.4)	74.1 (14.8)	±5	−3.8 (− 5.5 to − 2.2)^‡^	1.4 (− 0.4 to 3.2)^†^	−5.2 (− 7.0 to − 3.5)^‡^
Environmental	71.8 (14.3)	68.2 (14.6)	68.7 (12.8)	±5	− 2.7 (− 4.2 to − 1.1)^†^	−1.2 (− 2.9 to 0.4)^†^	−1.4 (− 3.1 to 0.2)^†^

^a^Approximately 0.3 SD

^b^English minus Chinese, English minus Malay, and Chinese minus Malay, adjusted for age, sex, education, marital status, smoking status, chronic disease and body mass index

†Equivalence confirmed; ‡Equivalence inconclusive

For the second set of analyses to rule out unobserved cultural differences (Table 3), when comparing the English and Chinese language versions of WHOQOL-BREF, confirmed equivalence was demonstrated for all domains. When comparing the English and Malay language versions, confirmed equivalence was demonstrated for all domains except the environmental domain, where equivalence was inconclusive.

**Table 3:** WHOQOL-BREF scales: mean (SD) by language version, equivalence margin and adjusted difference between the two language versions for only ethnic Chinese and ethnic Malay participants

		Ethnic Chinese^c^	Ethnic Malay^d^
Scale	Equivalence Margin^a^	English vs Chinese Difference^b^ (90% CI)	English vs Malay Difference^b^ (90% CI)
Physical	±5	−0.73 (−2.94 to 1.48)^†^	− 0.39 (− 3.12 to 2.33)^†^
Psychological	±5	−2.37 (− 4.73 to − 0.02)^†^	− 2.59 (− 4.98 to − 0.20)^†^
Social	±5	−1.76 (− 3.86 to 0.35)^†^	− 1.99 (− 4.58 to 0.59)^†^
Environmental	±5	−1.32 (− 3.53 to 0.89)^†^	− 2.90 (− 5.17 to − 0.63)‡

^a^Approximately 0.3 SD

^b^English minus Chinese, English minus Malay, adjusted for age, sex, education, marital status, smoking status, chronic disease and BMI

^c^Data from 172 ethnic Chinese participants completed the English version questionnaire and 427 ethnic Chinese participants completed the Chinese version questionnaire

^d^Data from 114 ethnic Malay participants completed the English version questionnaire and 345 ethnic Malay participants completed the Malay version questionnaire

^†^Equivalence confirmed; ^‡^Equivalence inconclusive

## Discussion

In this study, we assessed the 90% CI of the WHOQOL-BREF score differences between three language versions (English, Chinese and Malay) against a MID of 0.3 SD suggested by Farivar et al. [31]. The findings were slightly different depending on whether unobserved cultural differences were accounted for. There are two possible explanations for this. First, unobserved cultural differences may indeed have some effect on the estimations. Second, the set of analyses that accounted for unobserved cultural differences could be unstable due to a smaller number of participants completing the English version (172 ethnic Chinese or 114 ethnic Malay versus 428 all ethnicities). Both analyses fell short of the required sample size of 175. Robust conclusions regarding definite equivalence can thus only be drawn for two domains in the English and Chinese language versions, namely physical and environmental and for all domains in the English and Malay versions except environmental. To better understand if there are genuine differences in how the various ethnicities interpreted the items, further qualitative research or cognitive interviews will be useful.

A strength of this study is that we simultaneously examined three language versions, and these are the three major languages spoken in Singapore. By administering the three language versions using the same set of procedures, the design removed potential confounders and non-comparability between different studies that used different language versions. There are many factors that may affect comparability of study findings. For example, if the studies were conducted at different time periods, sporadic events such as haze due to slash-and-burn farming season in the neighbouring countries, may influence how individuals perceive their HRQoL. In addition, patient experience at the clinics may also influence their HRQoL. Hence, studies conducted in different clinic settings may yield different results. Therefore, by administering the three language versions within the same study using the same set of procedures and in the same settings may help to reduce some of these potential confounders and non-comparability. By over-sampling the Malay and Indian population, the design achieved sufficient precision to assess each language version, thus allowing for comparisons among the three versions. Hence, the conclusion on equivalence between language versions is generalizable to the population. Furthermore, we used a relatively stringent criteria for equivalence by using a MID of 0.3 SD [31]. However, there are potential limitations of the study. The WHOQOL-BREF is designed to be self-administered but in this study, the questionnaire was administered through face-to-face interviews. It has been shown that different modes of survey administration might result in different degree of true responses [34]. However, the bias is consistent throughout all the questionnaires and should not affect the analysis done across the various language versions, which is largely comparative. In this analysis, we accounted for potential confounders including age, sex, education, marital status, smoking status, chronic disease and BMI as these were the information available in our studies. Hence, there may be residual confounding such as those associated with level of physical activities and dietary quality.

## Conclusion

Using a MID of 0.3 SD, we found that the English and Chinese language versions were definitely equivalent for two of four domains. The English and Malay language versions of the WHOQOL-BREF were equivalent for all four domains except environmental. Since none of the domains were clearly not equivalent, it is reasonable to assume that the three language versions of the WHOQOL-BREF may be treated as equivalent. The scores from these language versions may thus be pooled together in the analyses and this will greatly increase the statistical power of the analyses.

## Abbreviations

ANOVA: Analysis of Variance
BMI: Body Mass Index
CI: Confidence Interval
HrQoL: Health-related Quality of Life
MID: Minimally Important Difference
NHCS: National Heart Centre Singapore
NUH: National University Hospital
PRO: Patient reported Outcomes
SD: Standard Deviation
STEMI: acute myocardial infarction
WHOQOL-BREF: World Health Organization Quality of Life Questionnaire
